# Identification of miRNA-Based Signature as a Novel Potential Prognostic Biomarker in Patients with Breast Cancer

**DOI:** 10.1155/2019/3815952

**Published:** 2019-12-30

**Authors:** Jia Tang, Wei Ma, Qinlong Zeng, Jieliang Tan, Keshen Cao, Liangping Luo

**Affiliations:** ^1^Medical Genetics Center, Jiangmen Maternity and Child Health Care Hospital, Jiangmen, Guangdong 529000, China; ^2^Department of Medical Imaging Center, The First Affiliated Hospital of Jinan University, Jinan University, Guangzhou, Guangdong 510080, China; ^3^Department of Biology, School of Basic Medicine, Jiamusi University, Jiamusi, Heilongjiang 154007, China

## Abstract

To identify the novel, noninvasive biomarkers to assess the outcome and prognosis of breast cancer (BC), patients with high sensitivity and specificity are greatly desired. Herein, the miRNA expression profile and matched clinical features of BC patients were extracted from The Cancer Genome Atlas (TCGA) database. The preliminary candidates were screened out by the univariate Cox regression test. Then, with the help of LASSO Cox regression analysis, the hsa-let-7b, hsa-mir-101-2, hsa-mir-135a-2, hsa-mir-22, hsa-mir-30a, hsa-mir-31, hsa-mir-3130-1, hsa-mir-320b-1, hsa-mir-3678, hsa-mir-4662a, hsa-mir-4772, hsa-mir-493, hsa-mir-556, hsa-mir-652, hsa-mir-6733, hsa-mir-874, and hsa-mir-9-3 were selected to construct the overall survival (OS) predicting signature, while the hsa-mir-130a, hsa-mir-204, hsa-mir-217, hsa-mir-223, hsa-mir-24-2, hsa-mir-29b-1, hsa-mir-363, hsa-mir-5001, hsa-mir-514a-1, hsa-mir-624, hsa-mir-639, hsa-mir-659, and hsa-mir-6892 were adopted to establish the recurrence-free survival (RFS) predicting signature. Referring to the median risk scores generated by the OS and RFS formulas, respectively, subgroup patients with high risk were strongly related to a poor OS and RFS revealed by Kaplan-Meier (K-M) plots. Meanwhile, receiver operating curve (ROC) analysis validated the accuracy and stability of these two signatures. When stratified by clinical features, such as tumor stage, age, and molecular subtypes, we found that the miRNA-based OS and RFS classifiers were still significant in predicting OS/RFS and showed the best predictive values than any other features. Besides, functional prediction analyses showed that these targeted genes of the enrolled miRNAs were enriched in cancer-associated pathways, such as MAPK/RTK, Ras, and PI3K-Akt signaling pathways. In summary, our observations demonstrate that the novel miRNA-based OS and RFS signatures are independent prognostic indicators for BC patients and worthy to be validated by further prospective studies.

## 1. Introduction

A sum of 268,600 new invasive breast cancer (BC) cases are estimated in the United States in 2019, with an approximate 41,760 BC-related deaths [1]; the average 5-year survival rate for women with invasive breast cancer is 90%, and the average 5-year overall survival (OS) of BC patients are different from 99% to 27% due to the different stages of pathological development. It is worthy of note that many factors including the size of the tumor, the number of lymph nodes that contain cancer, and other features of the cancer that affect how quickly cancer will grow and how well treatment works may have a crucial role in BC patients' OS and disease-free survival. Besides, Breast Cancer Index, OncotypeDX, Prosigna, EndoPredict, MammaPrint, Mammostrat, and IHC4 are also validated by clinical trials, some of which have already been approved by FDA and listed in the guidelines [2]. For example, the OncotypeDX model, a 21-gene signature, is regarded as one of the best-validated breast cancer multigene signatures, suggesting a stratification of the five-year or ten-year risk of distant relapse [3].

Recently, the detection of free circulating microRNAs (miRNAs) allows the establishment of multivariate models or signatures, which could be used to monitor the disease status and predict the prognosis of cancer patients. miRNAs are a class of noncoding RNA (ncRNA) molecules. As the single-strand RNA is small, they have recently emerged as pivotal gene expression regulatory molecules in the multicellular organism [4]. Accumulating evidence has shown that expression of the miRNA and its target genes is influenced at numerous levels, including epigenetic effects, promoter regulation, RNA processing and stability, and translation, having functional effects on cell proliferation, apoptosis, metastasis, and sensitivity to chemotherapy and radiotherapy in breast cancer [5]. Furthermore, studies have highlighted the significant value of miRNA in predicting prognosis of BC, especially for invasive BC [6]. The most well-known example is that overexpression of miR-21 and miR-210 results in shorter overall survival of BC patients [7]. The Cancer Genome Atlas (TCGA) provided us with complicated clinical characteristics and more than cancer genomics [8]. Screened by 1000 BC samples, we retrieved to make an understanding of the relationship of miRNA expression level with the prognosis and outcome of BC patients.

Herein, we applied the least absolute shrinkage and selection operator (LASSO) method aiming to develop two miRNA-based prognosis signatures for overall survival (OS) and recurrence-free survival (RFS) prediction, respectively, based on The Cancer Genome Atlas (TCGA) database [9–11].

## 2. Materials and Methods

### 2.1. Data Source from TCGA

Normalized read counts of miRNA expression profiles of 1098 BC patients in combination with clinicopathological and molecular information were obtained from the TCGA website in July 2019 (https://xenabrowser.net/datapages/?cohort=TCGA%20Breast%20Cancer%20(BRCA)&removeHub=https%3A%2F%2Fxena.treehouse.gi.ucsc.edu%3A443). And 1057 patients with available OS information and 886 patients with available RFS information were extracted for downstream analysis of OS-associated and RFS-associated marker detection, respectively. These patients were randomly arranged by 7 : 3 in the order of training cohort and validation cohort by a computer-generated allocation sequence for both OS- (740 vs. 317) and RFS-related (620 vs. 266) analyses, respectively.

### 2.2. Candidate OS/RFS-Relevant Gene Identification and Signature Generation

The univariate Cox regression test and Kaplan-Meier (K-M) survival analysis were executed to screen out the potential OS/RFS-relevant miRNAs. A *P* value less than 0.05 was considered statistically significant. Then, we applied the LASSO Cox regression test and Cox's proportional hazards (HRs) to find out the key miRNAs. The regularization parameter *λ* determined by the crossvalidated standard error (SE) introduced the penalties of the model establishing the process to avoid overfitting and the larger *λ* [12]. Then, a list of miRNAs was eventually picked up, of which *β*‐coefficients > 0. Finally, the risk score formulas composed of the sum values of miRNA expression weighted by the multivariate Cox regression coefficients were obtained. The median risk score was set as the cutoff point, according to which the BC patients were assigned into high- and low-risk subgroups.

### 2.3. Evaluation of the miRNA-Based Risk-Predictive Models

The K-M plot was employed to verify the survival difference between high- and low-risk populations. The area under the curve (AUC) of the receiver operating characteristic (ROC) curve was employed to determine the efficiency and accuracy of the OS/RFS classifier. Moreover, to confirm whether the OS and RFS signatures are independent prognostic markers of BC patients, we performed stratified analysis by different clinical features (gender, age, tumor grade, new tumor event after initial treatment, and molecular subtypes).

### 2.4. Functional Annotation of Identified miRNAs

We obtained potential target genes of prognostic miRNAs for BC OS/RFS-relevant miRNAs from TargetScan, miRTarBase, and miRDB databases [13–15]. Cytoscape software was used for the visualization of the miRNA-target network to understand miRNA function and regulatory mechanisms. Besides, we further performed the Kyoto Encyclopedia of Genes and Genomes (KEGG) pathway, the Gene Ontology (GO), and the Reactome analyses for miRNA-targeted genes.

## 3. Results

### 3.1. Identification of OS/RFS-Related miRNA and Construction of an miRNA-Based Prognostic Model

**Figure 1:**
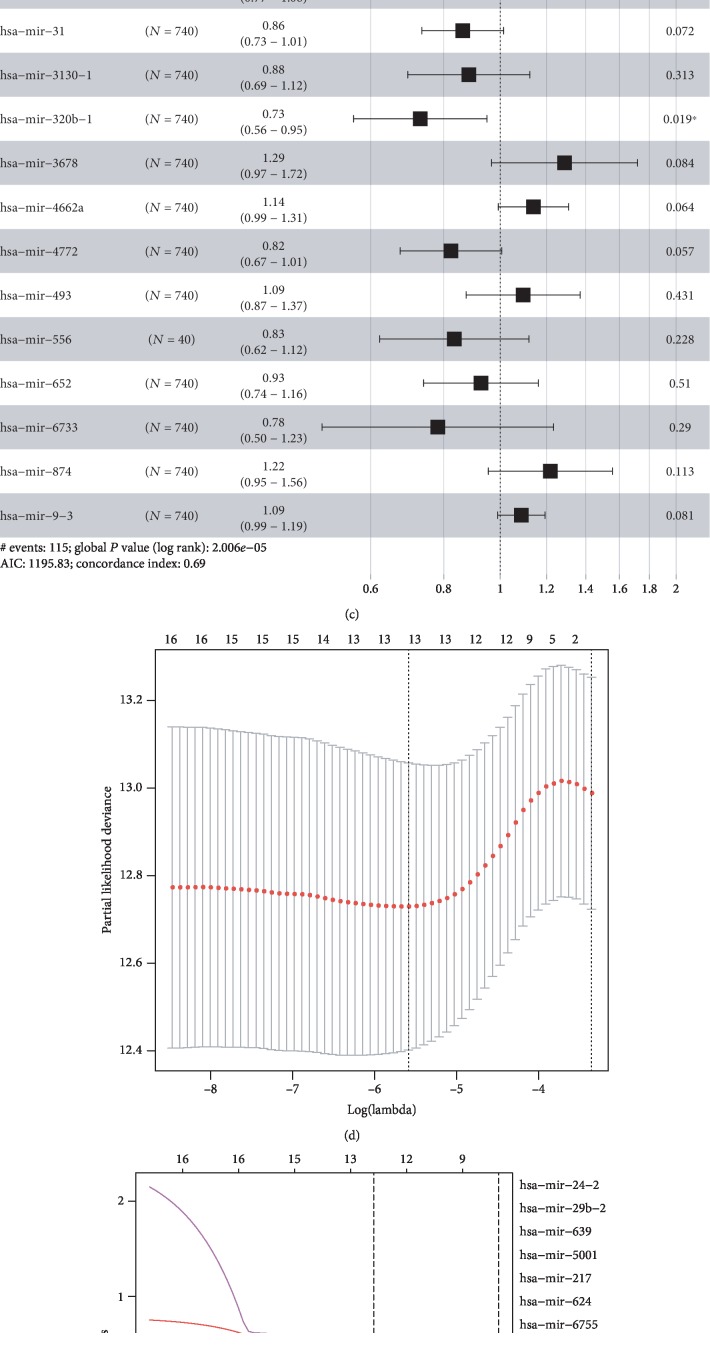
Construction of the OS/RFS miRNA signatures. Tuning parameter (*λ*) selection crossvalidation error curve for OS/RFS-relevant miRNA. The vertical lines were drawn at the optimal values by the minimum criteria and the 1-SE criteria (a, d). The LASSO coefficient profiles of 17 overall survival-related miRNAs; the vertical line is drawn at the value chosen by 10-fold crossvalidation (b). The LASSO coefficient profiles of 13 recurrence-free survival-related miRNAs; the vertical line is drawn at the value chosen by 10-fold crossvalidation (e). Hazard ratio of the enrolled OS-related miRNAs (c) and enrolled RFS-related miRNAs (f). LASSO coefficient profiles of OS-relevant miRNAs associated with the prognosis of patients with breast cancer (a–c); LASSO coefficient profiles of RFS-relevant miRNAs associated with the prognosis of patients with breast cancer (d–f).

We enrolled a total of 1098 BC patients, with available miRNA expression data from TCGA database. For OS-related miRNA detection, 1057 patients with available OS information were extracted and were randomly separated into a training set (*n* = 740) and validation set (*n* = 317) (Table 1). For RFS-related analysis, 886 patients with available RFS information were extracted and were randomly sorted into a training set (*n* = 620) and validation set (*n* = 266) (Table 2). The univariate Cox regression analysis revealed 23 OS-related miRNAs (*P* < 0.05) ([Supplementary-material supplementary-material-1]) and 20 RFS-related miRNAs ([Supplementary-material supplementary-material-1]). Of the miRNA profile in Figures 1(a)–1(b), a set of 17 OS-relevant miRNAs with the coefs were applied to establish the risk score formula of OS. Herein, we constructed a 17-miRNA-based OS classifier as follows. Risk score = −0.041∗let‐7b + 0.009∗miR‐101‐2 − 0.066∗miR‐135a‐2 + 0.212∗miR‐22 − 0.100∗miR‐30a − 0.148∗miR‐31 − 0.124∗miR‐3130‐1 − 0.315∗miR‐320b‐1 + 0.253∗miR‐3678 + 0.131∗miR‐4662a − 0.194∗miR‐4772 + 0.090∗miR‐493‐0.181∗miR‐556‐0.076∗miR‐652 − 0.246∗miR‐6733 + 0.198∗miR‐874 + 0.083∗miR‐9‐3 (Figure 1(c) and [Supplementary-material supplementary-material-1]). For RFS-related miRNAs, LASSO-penalized Cox analysis was performed to draw out the predicting signature of the 13-miRNA-based RFS classifier (Figures 1(d) and 1(e)), the risk score formula = −0.313∗miR‐130a − 0.113∗miR‐204 + 0.370∗miR‐217‐0.226∗miR‐223 + 0.597∗miR‐24‐2 − 0.047∗miR‐29b‐1 − 0.128∗miR‐363 + 0.447∗miR‐5001 − 0.275∗miR‐514a‐1 + 0.280∗miR‐624 + 0.474∗miR‐639‐0.461∗miR‐659‐0.268∗miR‐6892 (Figure 1(f) and [Supplementary-material supplementary-material-1]). In Figure 1(f), we identified five miRNA genes (miR-217, miR-24-2, miR-5001, miR-624, and miR-639) that have a positive impact on the RFS of BC patients, as well as the other eight miRNAs, including miR-130a, miR-204, miR-223, miR-29b-1, miR-363, miR-514a-1, miR-659, and miR-6892, which were negatively correlated with the recurrence of BC patients.

### 3.2. Verification of the miRNA Signatures in Training and Validation Sets

**Figure 2:**
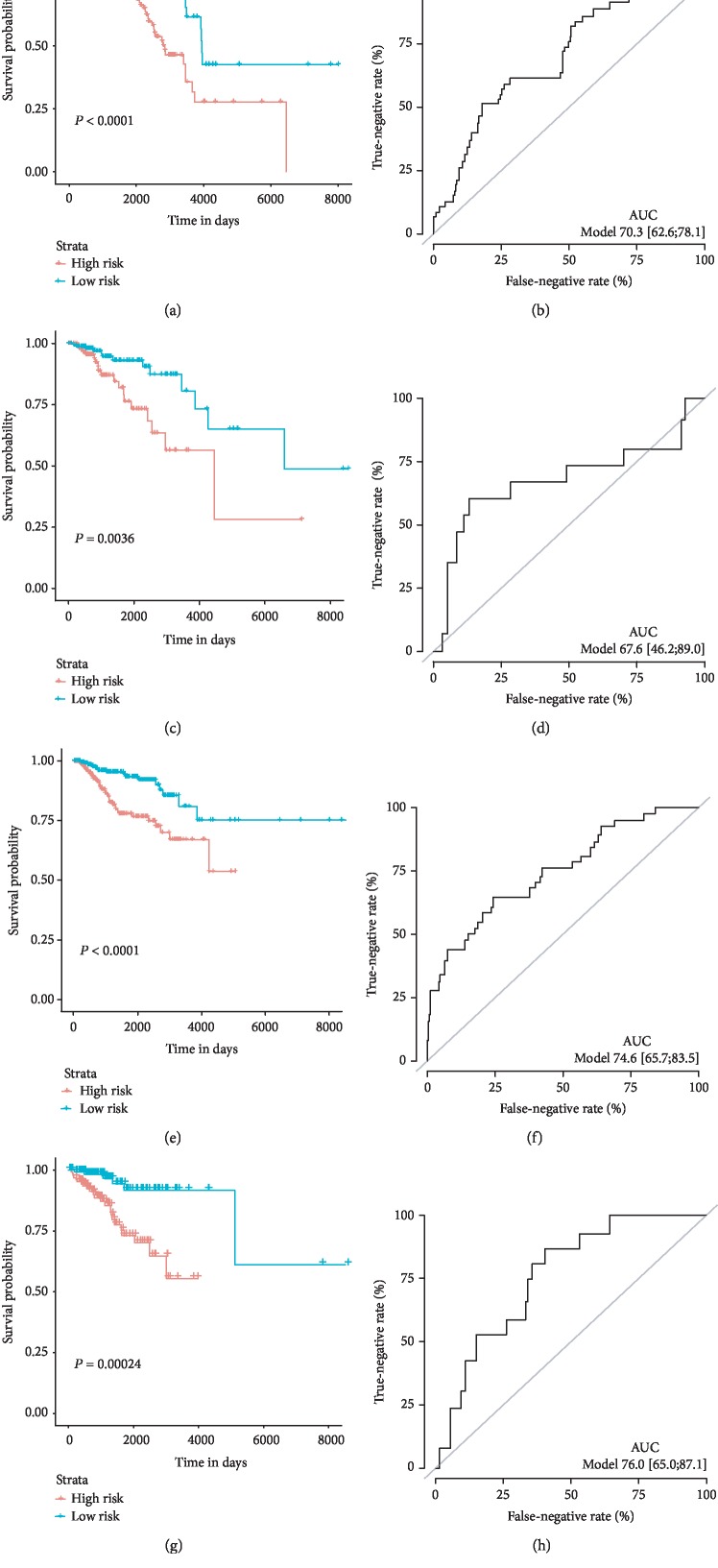
OS/RFS miRNAs predict the signature performance in breast cancer patients. Kaplan-Meier curves of the low- and high-risk groups divided by the 17-miRNA-based OS-predictive signature in the training cohort (a) and validation cohort (c). Kaplan-Meier curves of the low- and high-risk groups divided by the 13-miRNA-based RFS-predictive signature in the training cohort (e) and validation cohort (g). ROC curves of the low- and high-risk groups divided by the 17-miRNA-based OS-predictive signature in the training cohort (b) and validation cohort (d). ROC curves of the low- and high-risk groups divided by the 13-miRNA-based RFS-predictive signature in the training cohort (f) and validation cohort (h).

The risk score could be obtained by the multiplication of the regression coefficient of each selected miRNA and the normalized expression results derived from each patient, and we then ranked these patients referring to the risk scores. As such, high-risk and low-risk groups were distributed according to the median risk score. The K-M curves were applied to compare the OS of the two subgroups. The results showed that the high-risk patients have a remarkably shorter OS than the low-risk patients (*P* < 0.0001, Figure 2(a)) in both the training and validation sets (*P* < 0.0001, Figure 2(c)). In ROC analysis to assess the accuracy of the 17-miRNA-based signature, the AUC values at 5-year survival achieved 0.703 (95% CI: 0.628-0.781) and 0.746 (95% CI: 0.657-0.835) in the training cohort and validation cohort, respectively (Figures 2(b) and 2(d)), showing favorable discrimination for BC patients. For the 13-miRNA-based RFS classifier, significant differences were exhibited between the two groups in both the training and validation sets (*P* = 0.0036, Figure 2(e); *P* = 0.00024, Figure 2(g)). And the AUC of the ROC curves of RFS was 0.676 in the testing set (Figure 2(f)) and 0.760 in the validation set (Figure 2(h)).

### 3.3. Functional Enrichment Analysis of the Target Gene

**Figure 3:**
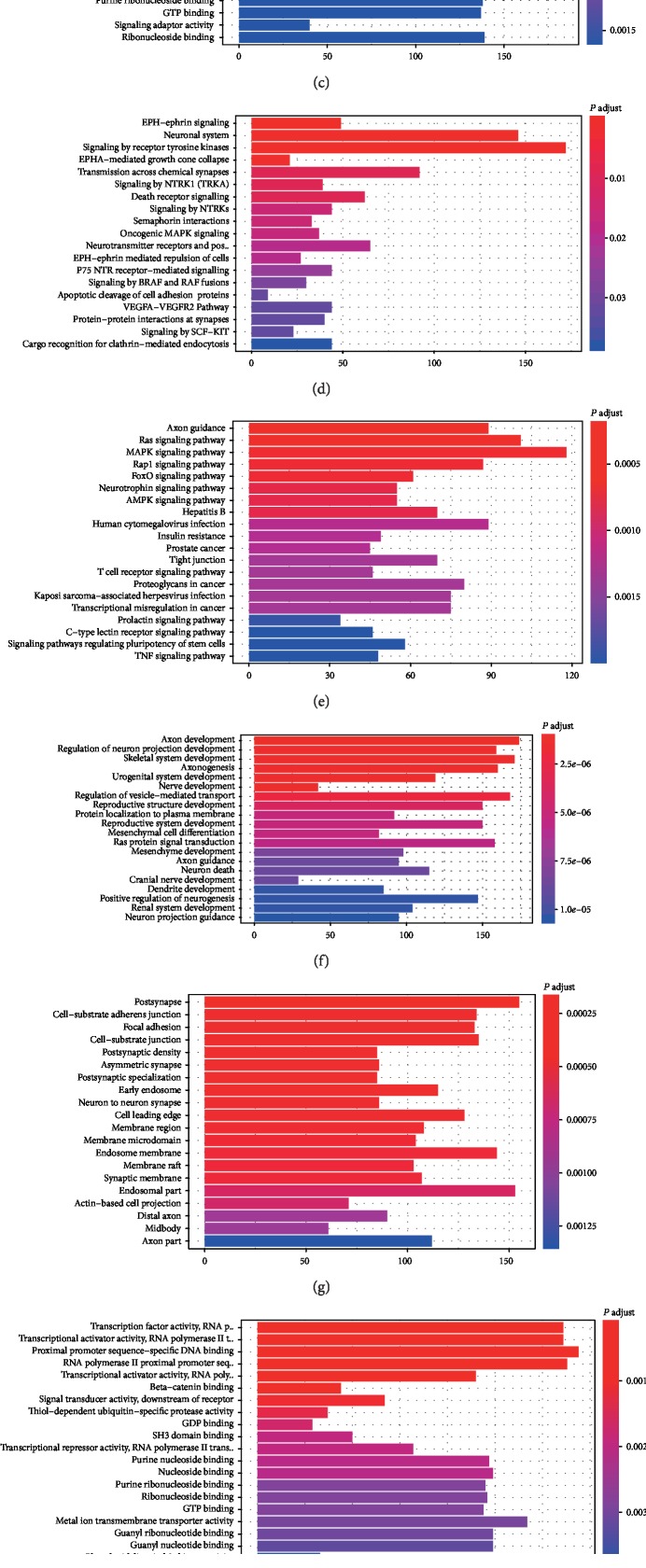
Functional enrichment analysis depicted the biological pathways and processes associated with OS/RFS-correlated genes. The results of GO-BP biological process enrichment (a), GO-CC biological process enrichment (b), GO-MF biological process enrichment (c), hallmark biological process enrichment (d), KEGG signaling pathway analysis (e), Reactome biological process enrichment (f).

To evaluate the potential function of target miRNA in the OS/RFS signatures, the miRNA-miRNA network for the downstream genes of the miRNAs related to the OS/RFS classifier was screened and displayed in [Supplementary-material supplementary-material-1] with the visualization tool Cytoscape. We also summarized the OS/RFS-relevant genes into GO, KEGG, and Reactome analyses. The analyses revealed that target genes of different miRNAs were significantly enriched in many biological processes related to tumorigenesis and progression processes, such as regulation of cell morphogenesis, cell-substrate adherens junction, transcription factor activity, and pathways such as signaling by the receptor tyrosine kinase, Ras, and PI3K-Akt signaling pathway (Figure 3).

### 3.4. Multivariate Analyses and Subgroup Analysis

**Figure 4:**
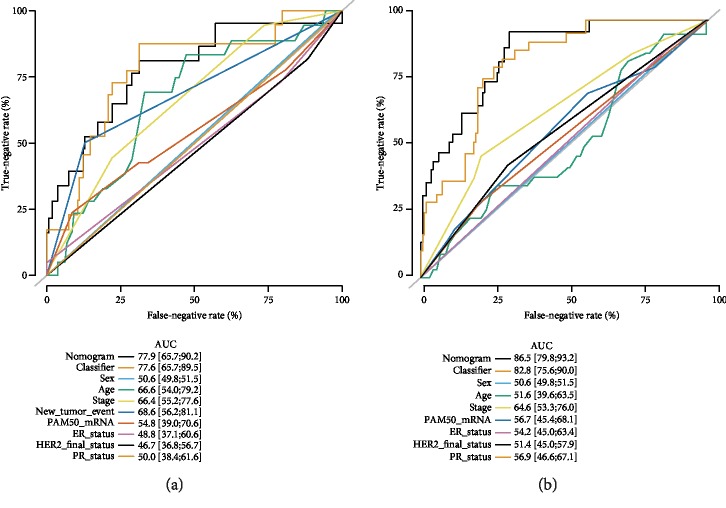
The comparison between the miRNA-based OS/RFS classifiers and clinical pathological features. (a) The hazard ratio of enrolled clinicopathological features and 17-miRNA-based OS classifiers in the overall set, respectively. (b) A hazard ratio of enrolled clinicopathological features and 13-miRNA-based RFS classifiers in the overall set, respectively.

Multivariate analyses confirmed that the OS and RFS signatures were the independent indicators for BC patients ([Supplementary-material supplementary-material-1] and [Supplementary-material supplementary-material-1]). We found that the predictive value of our signature was superior to the clinicopathological features, such as sex, age, tumor stage, new tumor event after initial treatment, and molecular subtypes alone. Then, nomograms were generated by combing our classifier and clinicopathological features, and the results indicated that the nomogram had the best predictive value, followed by our classifier (Figure 4).

**Figure 5:**
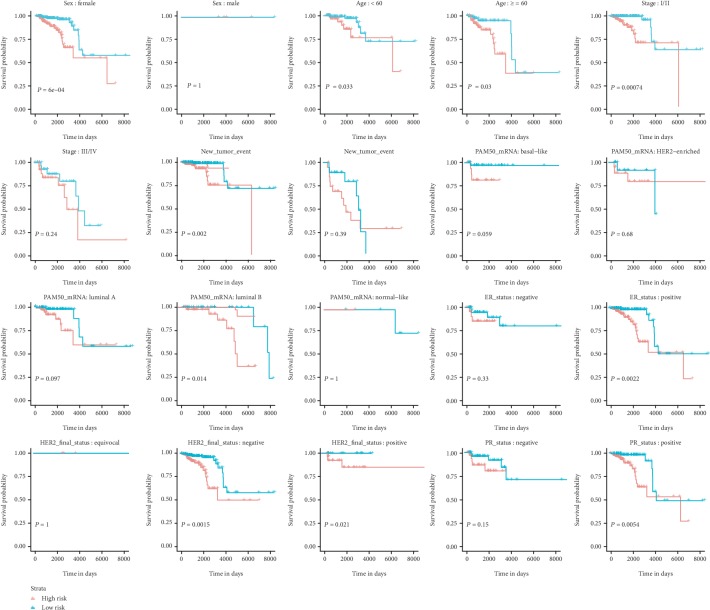
Subgroup analyses of the miRNA-based overall survival-related signature.

In addition, patients with complete information of gender, age, tumor grade, new tumor events after initial treatment, and molecular subtypes were included for further subgroup analysis. According to Figure 5, the OS classifier has independently predictive ability regardless of age (<60, ≥60). In the case of gender and tumor grade, the OS classifier showed more value in female (*P* = 6^×^10^−4^) and stage I/II patients (*P* = 0.00074) than in male (*P* = 1) and stage III/IV patients (*P* = 0.24), respectively. The miRNA-based OS predicting signature was still significant for these patients without new tumor events (*P* = 0.002). K-M curves of patients with different molecular subtypes demonstrated that the miRNA-based OS signature was more suitable for the risk prediction in luminal B (*P* = 0.014), positive ER status (*P* = 0.0022), negative HER2 status (*P* = 0.0015), and positive PR status (*P* = 0.0054) subgroups.

**Figure 6:**
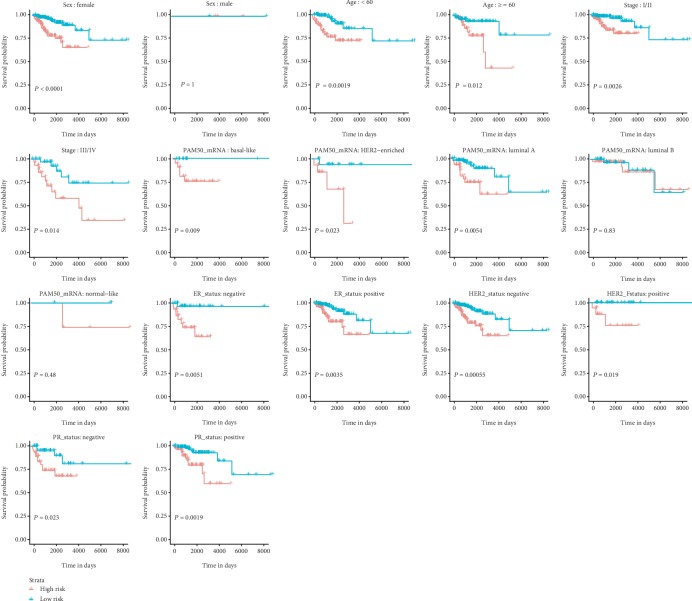
Subgroup analyses of the miRNA-based recurrence-free survival-related signature.

In addition, similar results were obtained for the miRNA-based RFS signature. As validation was performed on distinct ages (≥60 vs. <60) and the tumor stage (I/II vs. III/IV), the results showed the similar prognostic value (Figure 6). When adjusted by sex, the RFS signature remained to be an independent prognostic biomarker with restriction to female (*P* < 0.0001). The K-M curve analysis stratified by different molecular subtypes revealed remarkable discriminations in patients with different ER, HER2, and PR statuses. In respect of the intrinsic subtype of PAM50, reliability and general applicability for distinguishing each group were observed with basal-like (*P* = 0.009), HER2-enriched (*P* = 0.023), and luminal A (*P* = 0.0054) instead of luminal B and normal-like subgroups.

## 4. Discussion

The LASSO method is an innovative shrinkage and selection method for regression. Characterized as a high-dimensional predictor, it has been applied to classify the candidate miRNA genes and expression signature relevant to diagnosis and prognosis in the large dataset [16, 17]. In this study, we investigated and confirmed that the miRNA-based OS/RFS classifiers might potentially facilitate predicting the prognosis and clinical outcome of BC patients. Our results demonstrated that the OS model could distinguish the patients of BC with poor and good overall survival according to their risk score, and the 13-miRNA-based RFS classifier was robust to predict the outcomes of patients with BC. The ROC curve analysis of the two signatures revealed that the AUC was more significant than 0.7, which showed moderate predictive performance in the training cohort and validation cohort. Moreover, we also performed stratification analysis, and the results showed that the miRNA-based OS/RFS signature could be regarded as an independent prognostic factor of BC and patients in the high-risk group showed more reduced survival than patients in the low-risk group significantly after considering the various variables, such as gender, age and stages, and some molecular subtypes (ER status, HER2 status, and PR status). Furthermore, the result of GO and KEGG analyses revealed that the target genes of the OS/RFS signatures were involved in the related pathways such as Ras, MAPK, and PI3K-Akt signaling pathways, which were closely correlated with the differentiation, proliferation, migration, and invasion of the cancer cells [18, 19]. Besides, according to the functional analysis, the downstream genes might play critical roles during the regulation of cell morphogenesis, postsynapsis, and transcription factor activity, contributing to the mechanism description of the current work.

Generated by the LASSO regression method combining TCGA database, the miRNA-based prognosis model has been identified and verified in different tumors of recent researches [20–22]. Lai et al. [22] established a six-miRNA-based signature to predict the five-year OS in a small population of BC patients. In the study conducted by He et al. [23], they selected five miRNA candidates to establish the prognosis predicting signature. Although the signature has some impacts on the prognosis prediction, initially, these candidates were not strongly associated with the prognosis of BC patients revealed by the univariate Cox regression test. The further pathway enrichment analyses based on the targeted genes revealed the potential mechanisms of how these targeted genes influence the tumorigenesis and progression of BC. That is why we could not completely rely on the findings, enriched by the targeted genes of nonsignificant miRNAs. For the study conducted by Volinia et al. [24], they established and validated a miRNA signature clinically and their work provides critical guidance for clinical decision. It would be better if they pay slightly more attention to reveal the potential mechanisms of how these miRNAs or their targeted genes influence the fate of BC patients. In our work, we synthesized the advantages of these studies and achieved satisfied results.

In our work, a supervised OS miRNA signature is composed of 17 miRNAs, which have been investigated to result in the molecular characterization of several cancer types. Functions of the most relative miRNA such as let-7b, miR-101-2, miR-22, miR-30a, miR-31, miR-493, miR-652, miR-874, and miR-9-3 have been classified to be primarily attributed to the occurrence, development, and metastasis of BC. Besides, we also tested the expressions of these miRNAs (both OS- and RFS-related miRNAs) by comparing the high-risk and low-risk groups ([Supplementary-material supplementary-material-1]) and found that the tendency of their expression was consistent with the univariate Cox regression test. In the MDA-MB-231 and MDA-MB-468 cells, Zhang et al. [25] confirmed that the silencing of HOST2 induced cell proliferation inhibition and cell redistribution by the target miRNA of let-7b. Similarly, the let-7b miRNA gene was involved in the epithelial-to-mesenchymal transition process and cell growth in BC cells concerning Al-Harbi et al.'s report [26]. Li et al. [27] evaluated the clinical value of miR-101-2 for the prognosis and diagnosis of BC and concluded that downexpression of miR-101-2 could be utilized as a diagnostic marker, previously validated by a 1064-case-control study in 2014 [28]. miR-22 has been reported to play a dual role in the prognosis and recurrence of BC [23, 29, 30]. Damavandi et al. [31] firstly demonstrated a significant upregulation of miR-22 in breast cancer tissues through RT-PCR, and then, both Zou et al. and Liu et al.'s [32, 33] studies showed that aberrant expression of miRNA decreased the MCF-7 BC cell's ability to proliferate, migrate, and invade, facilitating our understanding of the molecular mechanism underlying the malignant behaviors of breast cancer cells. Unlike miR-22's protective role, downregulating miR-9-3 promotes breast cancer cell proliferation [34]. The impact of miR-30a and miR-31 on the initial and progress of BC seems to be deeply investigated than that in miR-22's studies. Other than the favorable biological functions such as inhibition of cell proliferation and migration [35, 36] and activation of the IGF1R/PI3K/AKT pathway [37], miR-31 influences the BC apoptosis by protein kinase C epsilon [38, 44] and regulates stem cell self-renewal and tumorigenesis by Wnt/*β*-catenin signaling [39], which is consistent with our enrichment analysis. Among the potential miRNA genes in the present study, we found that overexpression of miR-493 has been negatively correlated with monitoring the fidelity of chromosome segregation by mitotic arrest deficient-2 (MAD2), which predicts the efficacy of taxane chemotherapy [40]. For the miR-874 gene, some fundamental researches have already focused on predicting the tumor relapse and survival of BC patients [24, 41, 42].

With respect to the possible effects of the miRNAs on the RFS signature, a wide range of published literature has been investigated. miR-130a, a well-known tumor suppressor gene, is downregulated in human BC tissues and exosomes from circulating blood [43]. Higher expression levels of miR-130a can differentiate tumors from normal samples; also, higher expression levels of miR-130a can discriminate the molecular subtypes of breast cancer in a recent study [44]. miR-204-5p overexpression has a significant role in alteration in tumor metastasis and immune cell reprogramming by PI3K/Akt signaling in mouse breast cancer models [45]. miR-217 could suppress TNBC cell growth, migration, and invasion by downregulating the KLF5 expression [46]. Fabris et al. [47] revealed that patient radiotherapy (RT) after a lumpectomy has the poorer RFS as RT-induced miR-223 efficiently prevented BC cell growth by the EGFR pathway. Notably, through dampening cell survival, the overexpression of miR-24-2 reduces the growth of BC [48]. Also, miR-29b1 and miR-639 have been demonstrated to be informative biomarkers associated with the survival and recurrence of BC patients in related researches [49, 50]. The other three genes, miR-5001, miR-514a-1, and miR‐6892, have not been individually studied.

In conclusion, the current study shows novel miRNA-based OS and RFS prognostic models. Future large sample size, multicenter, and prospective clinical validations are warranted to verify these predictive tools before application in routine clinical practice.

## Figures and Tables

**Table 1:** The clinicopathological parameters of the overall survival-related training and validation sets.

Parameter	Training set (%) (*N* = 740)	Validation set (%) (*N* = 317)	Fisher's exact test (*P* value)	SMD
Sex			0.95	0.026
Male	9 (1.2)	3 (0.9)		
Female	731 (98.8)	314 (99.1)		
Age (year)			0.876	0.015
≤60	410 (45.4)	187 (56.2)		
>60	330 (44.6)	139 (43.8)		
Stage			0.828	0.085
I	128 (17.4)	50 (15.9)		
II	412 (56.1)	184 (58.6)		
III	169 (23.0)	72 (22.9)		
IV	16 (2.2)	4 (1.3)		
X	10 (1.4)	4 (1.3)		

Note: 12 patients of the breast cancer cohort (overall survival) were male.

**Table 2:** The clinicopathological parameters of the recurrence-free survival-related training and validation sets.

Parameter	Training set (%) (*N* = 620)	Validation set (%) (*N* = 266)	Fisher's exact test (*P* value)	SMD
Sex			1	0.015
Male	8 (1.3)	3 (0.1)		
Female	612 (98.7)	263 (98.9)		
Age (year)			0.904	0.014
≤60	354 (57.1)	150 (56.4)		
>60	266 (42.9)	116 (43.6)		
Stage			0.117	0.208
I	113 (18.3)	41 (15.5)		
II	357 (58.0)	146 (55.3)		
III	128 (20.8)	72 (27.3)		
IV	7 (1.1)	4 (1.5)		
X	11 (1.8)	1 (0.4)		

Note: 11 patients of the breast cancer cohort (overall survival) were male.

## Data Availability

No data should be uploaded in the current work.

## Supplementary Materials

Supplementary Materials Table S1: coefficient of each miRNA candidate for the miRNA-based OS predicting signature. Table S2: coefficient of each miRNA candidate for the miRNA-based RFS predicting signature. Table S3: multivariate analysis comparison between the OS classifier and clinical features. Table S4: multivariate analysis comparison between the RFS classifier and clinical features. Figure S1: Kaplan-Meier curves of survival-associated miRNA detected by univariable Cox regression analysis. (A) Overall survival-related miRNAs; (B) recurrence-free survival-related miRNAs. Figure S2: prediction of miRNA downstream target genes. (A) The downstream gene prediction of OS-related miRNAs: let-7b, miR-101-2, miR-135a-2, miR-22, miR-30a, miR-31, miR-3130-1, miR-320b-1, miR-3678, miR-4772, miR-4662a, miR-493, miR-556, miR-652, miR-6733, miR-874, and miR-9-3; (B) downstream gene prediction of RFS-related miRNAs: miR-130a, miR-204, miR-217, miR-223, miR-24-2, miR-29b-1, miR-363, miR-5001, miR-514a-1, miR-624, miR-639, miR-659, and miR-6892. Figure S3: the relative expression of overall survival- and recurrence-free survival-related miRNAs between the high-risk and low-risk populations.
