# Supplementary material for: Identification of miRNA-Based Signature as a Novel Potential Prognostic Biomarker in Patients with Breast Cancer
Source: Dis Markers. 2019 Dec 30;2019:3815952. doi: 10.1155/2019/3815952 (PMC6954483; doi:10.1155/2019/3815952)
Supplement: Supplementary Materials — Table S1: coefficient of each miRNA candidate for the miRNA-based OS predicting signature. Table S2: coefficient of each miRNA candidate for the miRNA-based RFS predicting signature. Table S3: multivariate analysis comparison between the OS classifier and clinical features. Table S4: multivariate analysis comparison between the RFS classifier and clinical features. Figure S1: Kaplan-Meier curves of survival-associated miRNA detected by univariable Cox regression analysis. (A) Overall survival-related miRNAs; (B) recurrence-free survival-related miRNAs. Figure S2: prediction of miRNA downstream target genes. (A) The downstream gene prediction of OS-related miRNAs: let-7b, miR-101-2, miR-135a-2, miR-22, miR-30a, miR-31, miR-3130-1, miR-320b-1, miR-3678, miR-4772, miR-4662a, miR-493, miR-556, miR-652, miR-6733, miR-874, and miR-9-3; (B) downstream gene prediction of RFS-related miRNAs: miR-130a, miR-204, miR-217, miR-223, miR-24-2, miR-29b-1, miR-363, miR-5001, miR-514a-1, miR-624, miR-639, miR-659, and miR-6892. Figure S3: the relative expression of overall survival- and recurrence-free survival-related miRNAs between the high-risk and low-risk populations. [file 3815952.f1.pdf]

Supplementary Information

Title page

Identification of miRNA-based signature as a novel potential prognostic biomarker in patients with breast cancer

Jia Tang[1,2];

tony2081129@163.com

Wei Ma[3];

mawei@westlake.edu.cn

Qinlong Zeng[1];

564916488@qq.com

Jieliang Tan[1];

18026882602@qq.com

Keshen Cao[1];

445730661@qq.com

Liangping Luo[2*]

tony2081129@sohu.com

1. Medical Genetics Center, Jiangmen Maternity and Child Health Care Hospital, Jiangmen, Guangdong, 529000, China;

2. Department of Medical Imaging Center, The First Affiliated Hospital of Jinan University, Jinan University, Guangzhou, Guangdong, 510080, China;

3. Department of Biology, School of Basic Medicine, Jiamusi University, Jiamusi, Heilongjiang, 154007, China

Correspondence to Liangping Luo

Address: Medical Imaging Center, the First Affiliated Hospital of Jinan University, Guangzhou, Guangdong, 510632, P R China.

Email: tony2081129@sohu.com

Tel.: +86 17727606698

Word counts: 5232 (Including the Reference)

Online Supplementary Materials

Supplementary Table 1-4 and Supplementary Figure 1-3

Fig. S1. Kaplan-Meier curves of survival associated miRNA detected with univariable Cox Regression analysis. A. Overall survival related-miRNAs; B. Recurrence-free survival related miRNAs.

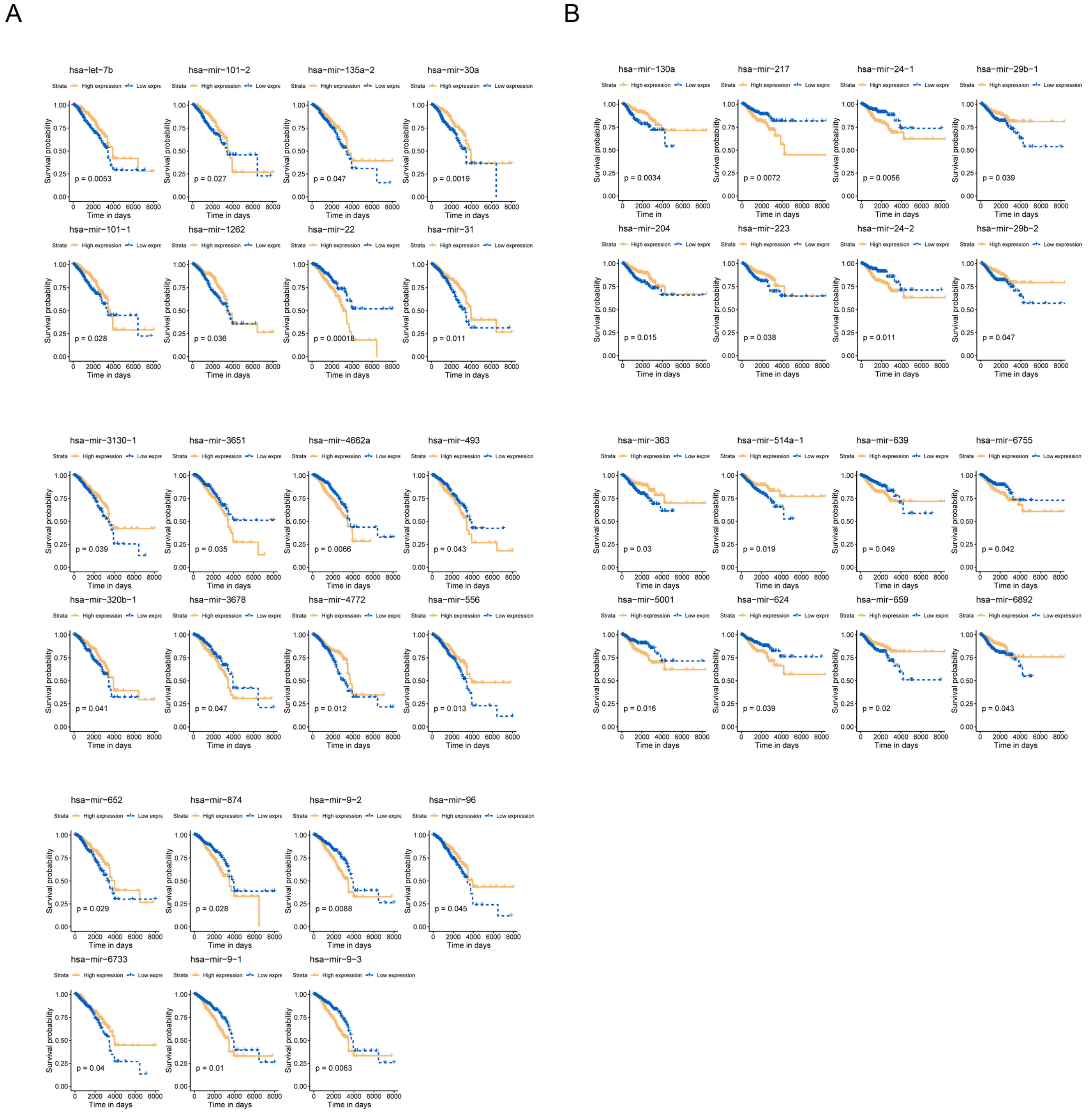

Figure S2. Prediction of miRNAs down-stream target genes. (A) The down-stream gene prediction of OS related miRNAs, let−7b, miR−101−2, miR−135a−2, miR−22, miR−30a, miR−31, miR−3130−1, miR−320b−1, miR−3678, miR−4772, miR−4662a, miR−493, miR−556, miR−652, miR−6733, miR−874, miR−9−3; (B) down-stream gene prediction of RFS related miRNAs, miR−130a, miR−204, miR−217, miR−223, miR−24−2, miR−29b−1, miR−363, miR−5001, miR−514a−1, miR−624, miR−639, miR−659, miR−6892.

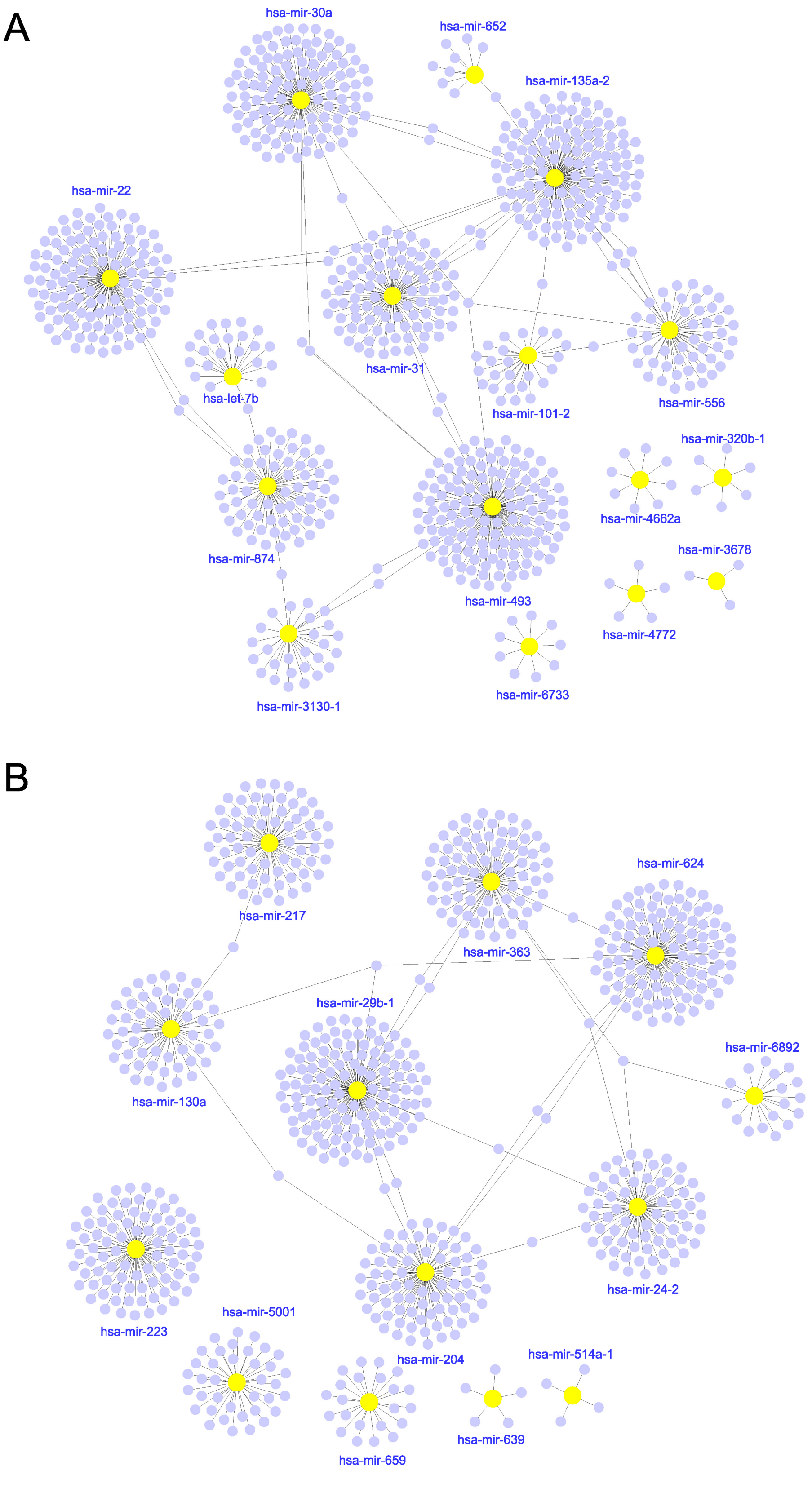

Figure S3. The relative expression of overall survival- and recurrence-free survival-related miRNAs between high-risk and low-risk populations.

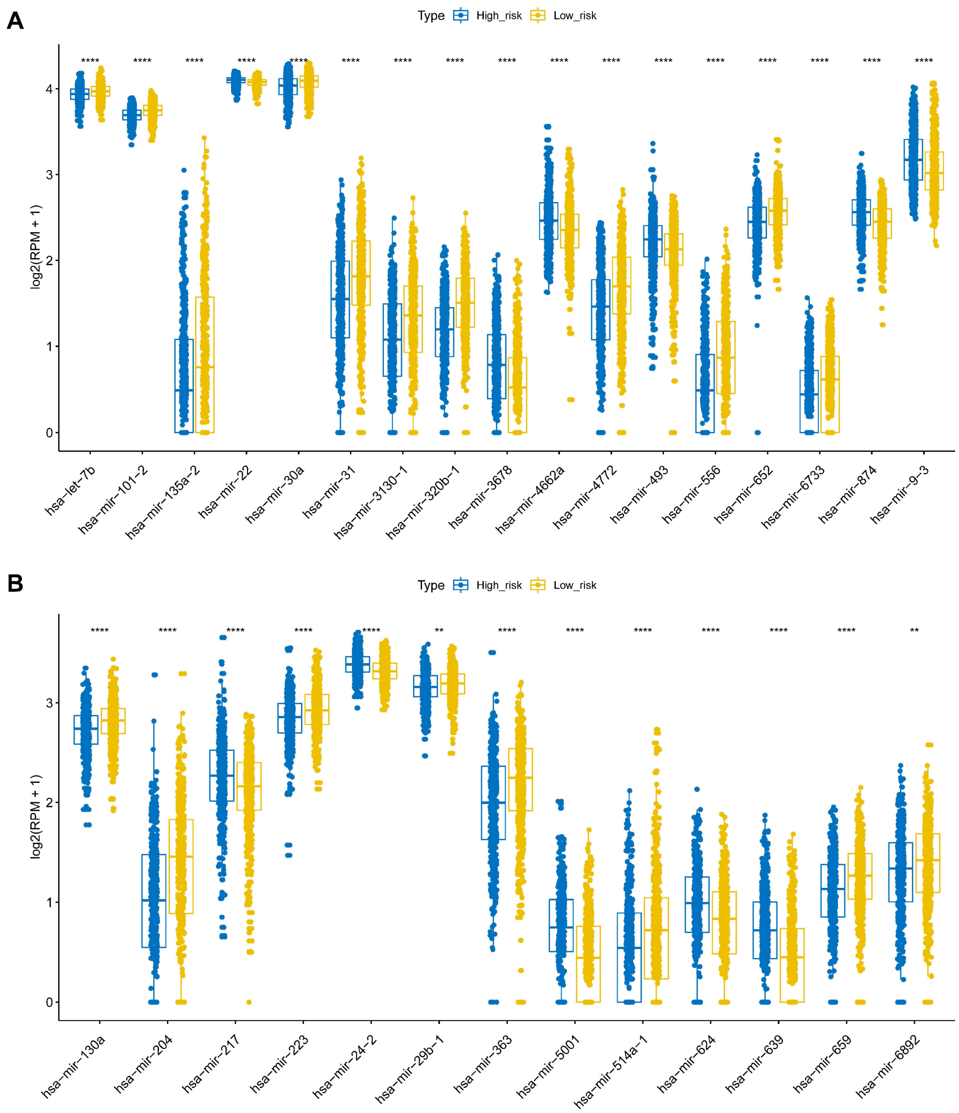

Table S1. Co-efficient of each miRNA candidate for the miRNA-based OS predicting signature

Gene ID	Co-ef	Exp (co-ef)	Se (co-ef)	z	*P*
hsa-let-7b	-0.04109	0.959742	0.119771	-0.34308	0.731539
hsa-mir-101-2	0.008913	1.008953	0.13396	0.066538	0.946949
hsa-mir-135a-2	-0.06609	0.936043	0.077752	-0.85006	0.39529
hsa-mir-22	0.212128	1.236306	0.210686	1.006845	0.314009
hsa-mir-30a	-0.10033	0.904536	0.082132	-1.2216	0.221858
hsa-mir-31	-0.14794	0.862485	0.082249	-1.79865	0.072074
hsa-mir-3130-1	-0.12391	0.883461	0.122755	-1.00939	0.312787
hsa-mir-320b-1	-0.31529	0.729578	0.134099	-2.35117	0.018714
hsa-mir-3678	0.253449	1.288461	0.146839	1.726032	0.084342
hsa-mir-4662a	0.130754	1.139687	0.070659	1.850484	0.064244
hsa-mir-4772	-0.19431	0.823403	0.102184	-1.90157	0.057227
hsa-mir-493	0.090304	1.094507	0.114672	0.787503	0.430987
hsa-mir-556	-0.18115	0.834309	0.150123	-1.20668	0.227554
hsa-mir-652	-0.07597	0.926841	0.115388	-0.65842	0.510269
hsa-mir-6733	-0.24615	0.781802	0.232751	-1.05759	0.290243
hsa-mir-874	0.197554	1.218418	0.124795	1.583026	0.113416
hsa-mir-9-3	0.08307	1.086618	0.047662	1.742916	0.081348

Co-ef, co-efficient; Exp (co-ef), Experiment (co-ef); Se (co-ef), standard error (co-efficient)

Table S2. Co-efficient of each miRNA candidate for the miRNA-based RFS predicting signature

Gene ID	Co-ef	Exp (co-ef)	Se (co-ef)	z	*P*
hsa-mir-130a	-0.313	0.731507	0.140419	-2.22654	0.025978
hsa-mir-204	-0.113	0.892711	0.132269	-0.85804	0.39087
hsa-mir-217	0.37	1.448053	0.118008	3.137242	0.001705
hsa-mir-223	-0.226	0.798087	0.126361	-1.78486	0.074284
hsa-mir-24-2	0.597	1.815876	0.179394	3.325472	0.000883
hsa-mir-29b-1	-0.047	0.954317	0.14927	-0.31326	0.754086
hsa-mir-363	-0.128	0.879506	0.08995	-1.42741	0.153462
hsa-mir-5001	0.447	1.564383	0.248224	1.802771	0.071424
hsa-mir-514a-1	-0.275	0.759493	0.182051	-1.51114	0.130752
hsa-mir-624	0.28	1.323083	0.227053	1.233034	0.217563
hsa-mir-639	0.474	1.60601	0.255213	1.856303	0.06341
hsa-mir-659	-0.461	0.63074	0.228269	-2.01894	0.043494
hsa-mir-6892	-0.268	0.764756	0.167808	-1.59824	0.109989

Co-ef, co-efficient; Exp (co-ef), Experiment (co-ef); Se (co-ef), standard error (co-efficient)

Table S3. Multivariate analyses comparison between OS classifier and clinical features

	Co-ef	Exp (co-ef)	Se (co-ef)	z	Pr (> |z|)
Sex (MALE)	-15.0323	2.96E-07	3824.367	-0.00393	0.996864
Age >= 60	1.213393	3.364883	0.386713	3.137712	**0.001703**
Stage III/IV	0.534275	1.70621	0.392192	1.36228	0.17311
New tumor event	1.468692	4.343551	0.389001	3.775552	**0.00016**
PAM50 mRNA (HER2-enriched)	1.701834	5.483997	0.879751	1.93445	0.053058
PAM50 mRNA (Luminal A)	-0.30345	0.73827	0.796881	-0.38079	0.703358
PAM50 mRNA (Luminal B)	0.517542	1.677898	0.818196	0.63254	0.527034
PAM50 mRNA (Normal-like)	1.264168	3.540147	1.183139	1.068487	0.285301
ER Status (Negative)	-3.47267	0.031034	1.467848	-2.36583	**0.01799**
ER Status (Positive)	-3.64004	0.026251	1.37128	-2.65448	**0.007943**
HER2 Status (Negative)	15.66269	6341932	8160.443	0.001919	0.998469
HER2 Status (Positive)	14.32124	1658191	8160.443	0.001755	0.9986
PR Status (Negative)	15.28017	4326080	3988.571	0.003831	0.996943
PR Status (Positive)	15.03724	3393048	3988.571	0.00377	0.996992
Classifier (High risk)	1.059404	2.884652	0.416962	2.54077	**0.011061**

Co-ef, co-efficient; Exp (co-ef), Experiment (co-ef); Se (co-ef), standard error (co-efficient); ER, estrogen receptor; PR, progesterone receptor

Table S4. Multivariate analyses comparison between RFS classifier and clinical features

	**Co-ef**	**exp(co-ef)**	**Se (co-ef)**	**z**	**Pr (> |z|)**
Sex (MALE)	-16.0902	1.03E-07	5750.799	-0.0028	0.997768
Age >= 60	0.211804	1.235905	0.366329	0.57818	0.563143
Stage III/IV	1.250033	3.490456	0.35243	3.546898	**0.00039**
PAM50 mRNA (HER2-enriched)	1.823381	6.19276	0.771997	2.3619	**0.018182**
PAM50 mRNA (Luminal A)	1.19249	3.295276	0.7128	1.672965	0.094334
PAM50 mRNA (Luminal B)	0.47467	1.607483	0.788836	0.601734	0.547351
PAM50 mRNA (Normal-like)	0.222358	1.249018	1.158583	0.191922	0.847803
ER Status (Negative)	16.94968	22969473	43567.37	0.000389	0.99969
ER Status (Positive)	17.02822	24846414	43567.37	0.000391	0.999688
HER2 Status (Negative)	18.26335	85441933	12803.39	0.001426	0.998862
HER2 Status (Positive)	16.69709	17842518	12803.39	0.001304	0.998959
PR Status (Negative)	17.79637	53563075	13006.94	0.001368	0.998908
PR Status (Positive)	16.77568	19301159	13006.94	0.00129	0.998971
Classifier (High risk)	1.4533	4.277207	0.378178	3.842905	**0.000122**

Co-ef, co-efficient; Exp (co-ef), Experiment (co-ef); Se (co-ef), standard error (co-efficient); ER, estrogen receptor; PR, progesterone receptor
